# Colorectal Cancer Awareness and Related Factors Among Adults Attending Primary Healthcare in North-Eastern of Iran: A Cross-sectional Study

**DOI:** 10.34172/jrhs.2023.124

**Published:** 2023-09-29

**Authors:** Samira Olyani, Hossein Ebrahimipour, Mehrsadat Mahdizadeh Taraghdari, Jamshid Jamali, Nooshin Peyman

**Affiliations:** ^1^Student Research Committee, Mashhad University of Medical Sciences, Mashhad, Iran; ^2^Department of Health Education and Health Promotion, School of Health, Mashhad University of Medical Sciences, Mashhad, Iran; ^3^Social Determinants of Health Research Center, Mashhad University of Medical Sciences, Mashhad, Iran; ^4^Department of Health Economic and Management Sciences, School of Health, Mashhad University of Medical Sciences, Mashhad, Iran; ^5^Department of Biostatistics, School of Health, Mashhad University of Medical Sciences, Mashhad, Iran

**Keywords:** Colorectal neoplasms, Awareness, Signs and symptoms, Risk factors, Cancer screening

## Abstract

**Background:** Colorectal cancer (CRC) is the third most prevalent cancer in Iran. This study aimed to assess the level of awareness regarding CRC warning signs, risk factors, screening program, and related factors among adults in North-Eastern Iran.

**Study Design:** A cross-sectional study.

**Methods:** The multi-stage sampling method was used to survey 2614 participants attending primary healthcare centers in Mashhad, Iran. The data collection tools were the demographics section and Bowel/ CRC Awareness Measure (Bowel/Colorectal CAM). The data were analyzed by SPSS, version 25. The significance level of the data analysis was less than 0.05.

**Results:** Mean awareness for CRC warning signs and CRC risk factors were 2.85±2.13 and 3.63±1.85, respectively. Most participants (97.2%) had no awareness of the CRC screening program. There was a significant association between marital status, education, job, income, and family history of CRC with awareness of warning signs (*P*<0.001); moreover, there was a significant association between age, education, job, income, and family history of CRC with awareness of risk factors (*P*<0.001). The results of logistic regression indicated that there was a significant association between age (*P*=0.022, OR=1.794, 95% confidence interval [CI]: 1.087, 2.962), gender (*P*=0.005, OR=0.488, 95% CI: 0.296, 0.803) and warning sign awareness (*P*<0.001, OR=1.278, 95% CI: 1.124, 1.454) with awareness of the CRC screening program.

**Conclusion:** In this study, most of the participants had low awareness of CRC. More aimed educational interventions are needed to promote Iranian adults’ awareness of CRC.

## Background

 Cancer is a significant health problem in both developed and developing countries around the world. In fact, it is the most important cause of morbidity and mortality which affects the socioeconomic status of the population.^1^

 Globally, colorectal cancer (CRC) ranks as the third most prevalent diagnosed cancer after lung and breast cancer and the second oncological cause of death with 1 931 590 new cases and 935 173 deaths in 2020.^2^ CRC is the second most common cancer in women and the third in men.^3^ Globally, CRC was estimated to cause 24.3 million (95% UI, 22.6-25 million) disability-adjusted life years in 2019; of these, 95.6% (95% UI, 94.4%-96.8%) came from years of life lost and 4.4% (95% UI,3.2%-5.6%) from years of life with a disability. In 2019, deaths due to CRC and incident CRC cases were 1.09 million (95% UI, 1.00-1.15 million) and 2.17 million (95% UI, 2.00-2.34 million), respectively.^4^

 In Iran, CRC with age-standardized incidence rate of 12.3 and 16.1 per 100 000 persons is the second and third most common cancer in women and men, respectively. In the north-east of Iran, CRC is the second most prevalent cancer in men and women with the age-standardized incidence rate of 18.24 and 13.96 per 100 000 persons, respectively. In Iran, a greater than 50% increase in the number of CRCs has been predicted by 2025.^5^

 The CRC is a highly preventable disease that requires public awareness of prevention and early diagnosis with available screening tests.^6^ It has been noted that the most important impediment to preventing this disease is lack of awareness regarding CRC.^7,8^ Inadequate awareness regarding CRC symptoms can be contributed to the manifestation and severity of the disease; moreover, it delays a patient’s presentation time which can worsen the outcomes.^9^ Considering the rising trend of CRC, raising awareness of CRC risk factors and symptoms could lead the general population to be more involved in primary preventive behaviors.^10^ Given that awareness of the risk factors and warning signs of CRC could lead to a decrease in the prevalence,^1^ it is necessary to perform primary preventive behaviors and plan to reduce the burden of this cancer.^11^ CRC is a multifactorial disease with different kinds of risk factors most of which (e.g., lack of physical activity, smoking, alcohol drinking, obesity, low fiber diet, high red meat consumption, consumption of processed meat, and low fruit and vegetable intake in the diet) are modifiable. Unmodifiable risk factors include genetics and age are just responsible for 10-15% of CRC cases.^5,8,11^ Studies indicated that about 95% of all CRC cases are discovered after presenting with symptoms such as bloody stool, unexplained weight loss, change in bowel habit, anemia, lower abdominal lump, rectal bleeding, rectal pain, and chronic abdominal pain which can be identified by patients.^1,10,12^

 CRC can be prevented with the detection and removal of polyps. Identifying patients with CRC at the early stages of the disease can lead to their survival.^13^ Adenomatous polyps which are in the premalignant state of CRC can be found and removed by screening. Moreover, being screened increases the probability of a cure with less expensive treatment.^14^

 An earlier study in northern Iran demonstrated that the awareness of CRC was poor among patients who referred to the hospital.^12^ The results of another study conducted on university staff also represented that about 50% of staff had a low level of awareness of CRC.^15^ Other studies in Middle East countries also reported low levels of CRC awareness.^16-19^

 Regarding the importance of CRC awareness, the primary objective of the current study was to assess the awareness of CRC among the adult population attending the primary healthcare centers in Mashhad. The secondary objective was to evaluate the differences in awareness among population subgroups.

## Methods

 This cross-sectional study was performed on participants visiting primary healthcare centers in Mashhad, Iran. The data were collected from June 2022 to September 2022. This article is part of a PhD thesis project, which was approved by the Ethics Committee of Mashhad University of Medical Sciences with the ethics code IR.MUMS.FHMPM.REC.1400.008 (Code: 991804). The sample size was calculated using the single population proportion formula with several assumptions, including a 95% confidence level, d/accuracy = 0.02, a hypothesis that 50% ( ± 5%) of participants were aware of CRC symptoms and risk factors,^1,17^ and a 10% sample loss rate. The sample size was estimated to be 2,640. The multi-stage sampling method was used to select and include the participants. Proportional stratified sampling and a simple random sampling method were employed in the first and second stages, respectively. In the proportional stratified sampling method, all health centers (n = 5) with their population were identified, and then each center was considered as a stratum. Next, the required sample size was selected from each center by simple random sampling from among those willing to participate in the study and who met the inclusion criteria for entry into the study. Finally, 518, 542, 2, 481, 538, and 561 people from different health centers (No. 1 to No. 5, respectively) entered the study.

 Completing the questionnaire was voluntary, all data were kept confidential, and an informed consent process was followed. To collect the data, the researcher referred to the health centers and explained the objectives of the study to the participants. All participants were informed about the objectives of the study. No incentives were offered to people for participation in the research, and they were free for quitting the study whenever they wanted. Participants who were eager to participate in the study filled out and signed a consent form before participation. The questionnaires were given to the participants who completed and signed the consent form. They completed the questionnaire in a quiet and secluded place in the health center. Further, the questionnaires for illiterate people were completed by the researcher.

 The inclusion criteria were being Iranian citizens and at least 18 years old, residing in Mashhad, and having no history of CRC, inflammatory bowel disease, or polyps; furthermore, completed written informed consent and willingness to participate in the study voluntarily were taken into consideration. Immigrants and citizens who did not reside in Mashhad were excluded from the study.

 The questionnaire is composed of two sections. The first section consists of questions investigating the demographic characteristics of the participants. Data related to age groups, gender, education level, job status, marital status, monthly income, and family history of CRC were collected in this section.

 The next section consists of questions that focus on ascertaining the awareness of participants regarding CRC. Data about CRC awareness were collected by utilizing a modified and translated-into-Persian version of the CAM. It was developed to assess awareness of cancer reliably among the general population by University College London and Cancer Research UK based on a generic CAM developed by Cancer Research UK, University College London, Kings College London, and Oxford University.^20^ Sections related to warning signs, risk factors, and screening were used in this study.

 In addition, the forward-backward translation concerning the cultural differences was employed, and the survey instrument (Bowel/colorectal CAM) was translated to Persian. The questionnaire was translated from English to Persian for the purpose of this study and then was back-translated into English. Each step was conducted by two independent bilingual healthcare professionals who were experts in clinical research and study design, none of whom were part of the research team.

 To ensure content validity, the questionnaire was then reviewed by ten independent experts in the fields of gastroenterology, health promotion, and public health (content validity ratio = 0.94).

 The face validity of 30 participants was measured before conducting the survey, and participants evaluated the comprehensiveness, clarity, and simplicity of the questionnaire; furthermore, the appropriateness and relevance of the items, the likelihood of ambiguity and misunderstandings, or any failure in conceptualization was determined by participants. In case of any problem, the participants’ comments were applied to the questionnaire.

 Internal consistency was evaluated using Cronbach’s alpha, which reached an acceptable value (α = 0.87). For assessing reliability, a test-retest was performed using data from (n = 30) those who completed the measure twice over two weeks (intraclass correlation coefficient = 0.92, *P* < 0.001).

 Awareness regarding CRC warning signs was assessed with questions phrased with “The following may or may not be warning signs for bowel cancer. We are interested in your opinion”. This is followed by the list of nine warning signs (bleeding from the back passage, persistent pain in the abdomen, change in bowel habit, feeling of incomplete emptiness of bowel, blood in stool, pain in the back passage, lump in the abdomen, tiredness/anemia, and unexplained weight loss) with response options of “Yes, No, and Do not know”. These questions are designed to measure how many warning signs a respondent can recognize when prompted. Each correct answer (yes) was given one point, and an incorrect answer (no or don’t know) was given a zero score. This gave a total awareness score ranging from 0 to 9.

 Awareness regarding CRC risk factors was evaluated with questions phrased with “These are some of the things that can increase a person’s chance of developing bowel cancer. How much do you agree that each of these can increase a person’s chance of developing bowel cancer?”. This is followed by a list of ten risk factors (drinking alcohol, smoking, eating less than 5 portions of fruit and vegetables a day, high intake of red meat, eating processed meat, having a diet low in fiber, being overweight or obese, being over 50 years old, having a close relative with bowel cancer, physical inactivity, having a bowel disease, and having diabetes. These questions are designed to measure a respondent’s level of agreement with the risk factors. Response options are “strongly disagree, disagree, not sure, agree, and strongly agree”. Each correct answer (agree or strongly agree) was given one point, and an incorrect answer (strongly disagree, disagree, or not sure) was given a zero score. The total awareness score ranged from 0 to 12. Mean awareness of warning signs and risk factors was reported finally.^17^

 Likewise, awareness regarding CRC screening was assessed by one question phrased with “As far as you are aware, is there a CRC screening program?” the response was “yes” or “no”.

###  Statistical Analysis

 Data were entered and analyzed using SPSS, version 25. In this study, data normality was performed using Kolmogorov-Smirnov test. Descriptive statistics were used to describe sample characteristics; moreover, the data were analyzed using multiple linear regression to determine the effect of various factors on the outcome variables. The degree of association between dependent and independent variables was assessed using odds ratio (OR) and coefficient with a 95% confidence interval and *P*-value. In the current study, univariate and multivariate binary logistic regressions were employed to investigate the association between the awareness of the CRC screening program as a dependent variable (yes/no) and demographic characteristics. First, each variable was entered into the univariate model, then each of them with a significance level of less than 0.2 (*P* < 0.2) was entered into the multivariate model.^21^ The significance level of the data analysis was less than 0.05.

## Results

 Out of 2640 distributed questionnaires, 2614 cases were completed, making a response rate of 99%, and 26 questionnaires were excluded from the study due to incomplete information. Finally, data analysis was performed on 2614 participants. The mean and standard deviation of the participants’ ages were 52.84 and 14.35. Most of the participants had a diploma degree or higher degrees (63.5%), and were employed (79.7%) and married (72.8%); furthermore, most of them had more than 170 USD monthly income (68.9%). Only 24.3% of participants (n = 634) reported having a family history of CRC. Out of all participants, 2540 cases (97.2%) had no awareness of the CRC screening program. The mean awareness of CRC warning signs was 2.85 ± 2.13. The most recognized warning sign was a lump in the abdomen (49.8%, n = 1301), while the least recognized warning sign was unexplained weight loss (11.7%, n = 305). The mean awareness of CRC risk factors was 3.63 ± 1.85. The most and least recognized risk factors were eating processed meat (82.7%, n = 2163) and high intake of red meat (5%, n = 132), respectively (Table 1).

**Table 1:** Demographic Characteristics and Awareness of Colorectal Cancer Warning Signs and Risk Factors (N = 2614)

**Characteristic**	**Number**	**Percent**
Age (y)		
< 50	1198	45.8
≥ 50	1416	54.2
Gender		
Male	1318	50.4
Female	1296	49.6
Marital status		
Single	710	27.2
Married	1904	72.8
Education		
< Diploma	955	36.5
≥ Diploma	1659	63.5
Job		
Employed/retired	2083	79.7
Unemployed	531	20.3
Income		
< 170 USD	812	31.1
≥ 170 USD	1802	68.9
Family history of CRC		
Yes	634	24.3
No	1980	75.7
CRC warning signs		
Bleeding from back passage	831	31.8
Abdominal pain	916	35
Change in bowel habit	775	29.6
Feeling of incomplete emptiness of bowel	644	24.6
Blood in stool	1300	49.7
Back passage pain	941	36
Lump in abdomen	1301	49.8
Tiredness/Anemia	461	17.6
Unexplained weight loss	305	11.7
CRC risk factors		
Alcohol consumption	1318	50.4
Smoking	757	29
Low intake of fruit/vegetables	195	7.5
High intake of red meat	132	5.0
Eating processed meat	2163	82.7
Low fiber diet	245	9.4
Being overweight or obese	780	29.8
Older age	732	28
Family history of bowel cancer	1151	44.0
Low physical activity	502	19.2
Having a bowel disease	1239	47.4
Having diabetes	287	11.0

*Note*. CRC: Colorectal cancer.

 The results demonstrated a significant association between marital status, education, job, income, and family history of CRC with awareness of warning signs (*P*< 0.001, Table 2).

**Table 2:** Association Between Awareness of Colorectal Cancer Warning Signs and Demographic Characteristic by Multiple Linear Regression (N = 2614)

**Variables**	**Unstandardized** **Coefficients of β**	**SE**	**Standardized** **Coefficients of β**	**t**	***P*****-value**	**95% CI for β**	
Constant	5.093	0.373		13.639	0.001	4.360	5.825
Age (y)							
< 50	1.000		1.000				
≥ 50	0.042	0.084	0.010	0.502	0.616	-0.122	0.207
Gender							
Male	1.000		1.000				
Female	0.114	0.084	0.027	1.362	0.173	-0.050	0.278
Marital status							
Single	1.000		1.000				
Married	-0.271	0.094	-0.056	-2.882	0.004	-0.455	-0.086
Education							
< Diploma	1.000		1.000				
≥ Diploma	1.443	0.082	0.325	17.561	0.001	1.282	1.604
Job							
Employed/retired	1.000		1.000				
Unemployed	-1.095	0.102	-0.206	-10.759	0.001	-1.294	-0.895
Income							
< 170 USD	1.000		1.000				
≥ 170 USD	0.417	0.090	0.094	4.637	0.001	0.241	0.594
Family history of CRC							
Yes	1.000		1.000				
No	-1.731	0.092	-0.347	-18.909	0.001	-1.910	-1.551

*Note*. CRC: Colorectal cancer; CI: Confidence interval.

 Based on the results, a significant association was found between age, education, job, income, and family history of CRC with awareness of risk factors (*P*< 0.001, Table 3).

**Table 3:** Association Between Awareness of Colorectal Cancer Risk Factors and Demographic Characteristic by Multiple Linear Regression (N = 2614)

**Variables**	**Unstandardized** **Coefficients of β**	**SE**	**Standardized** **Coefficients of β**	**t**	***P*****-value**	**95% CI for β**	
Constant	5.905	0.334		17.674	0.001	5.249	6.560
Age (y)							
< 50	1.000		1.000				
≥ 50	-0.528	0.072	-0.142	-7.325	0.001	-0.669	-0.387
Gender							
Male	1.000		1.000				
Female	0.051	0.073	0.014	0.706	0.480	-0.091	0.194
Marital status							
Single	1.000		1.000				
Married	0.108	0.082	0.026	1.318	0.187	-0.052	0.267
Education							
< Diploma	1.000		1.000				
≥ Diploma	0.825	0.074	0.214	11.210	0.001	0.681	0.969
Job							
Employed/retired	1.000		1.000				
Unemployed	-0.253	0.090	-0.055	-2.808	0.005	-0.429	-0.076
Income							
< 170 USD	1.000		1.000				
≥ 170 USD	0.347	0.078	0.087	4.545	0.001	0.194	0.500
Family history of CRC							
Yes	1.000		1.000				
No	-1.586	0.079	-0.367	-20.135	0.001	-1.740	-1.432

*Note*. CRC: Colorectal cancer; CI: Confidence interval.

 The results of logistic regression indicated that there was a significant association between age (*P*= 0.022, OR = 1.794, 95% CI: 1.087, 2.962), gender (*P*= 0.005, OR = 0.488, 95% CI: 0.296, 0.803), and warning sign awareness (*P*< 0.001, OR = 1.278, 95% CI: 1.124, 1.454) with awareness of CRC screening (Table 4).

**Table 4:** Association Between Awareness of Colorectal Cancer Screening Program and Demographic Characteristic by Logistic Regression (n = 2614)

**Variable**	**Awareness of CRC Screening **^a^					
	**Univariate Regression**			**Multivariable Regression**		
	**OR**	**95% CI**	***P*****-value**	**OR**	**95% CI**	***P***** value**
Age (y)						
< 50	1.000			1.000		
≥ 50	1.403	0.871-2.259	0.163	1.794	1.087-2.962	0.022
Gender						
Male	1.000					
Female	0.509	0.313-0.830	0.007	0.488	0.296-0.803	0.005
Marital status						
Single	1.000					
Married	0.939	0.563-1.569	0.811	-	-	-
Education						
< Diploma	1.000					
≥ Diploma	2.311	1.304-4.096	0.004	1.403	0.752-2.620	0.288
Job						
Employed/retired	1.000					
Unemployed	0.142	0.128-0.419	0.921	-	-	-
Income						
< 170 USD	1.000					
≥ 170 USD	1.415	0.826-2.422	0.206	-	-	-
Family history of CRC						
Yes	1.000					
No	0.458	0.285-0.734	0.001	0.666	0.393-1.127	0.130
Warning signs awareness^b^	1.335	1.185-1.504	0.001	1.278	1.124-1.454	0.001
Risk factors awareness^c^	0.920	0.810-1.046	0.204	**-**	**-**	-

*Note*. CRC: Colorectal cancer; CI: Confidence interval; OR: Odds ratio. ^a^The dichotomous dependent variable (Yes or No). ^b^Score between 0 and 9. ^c^Score between 0 and 12.

## Discussion

 The results of this study represented a low level of CRC warning signs, risk factors, and screening program awareness, which is in line with the results of other studies, demonstrating low levels of CRC awareness in various Asian countries, including India, Brunei, Malaysia, Singapore, Lebanon, Jordan, Qatar, United Arab Emirates, and Bahrain.^6,16-19,22,23^ Conversely, studies in Western countries reported good CRC awareness.^20,24-27^ In comparison to our study, a population survey in the UK using the bowel/colorectal CAM instrument showed higher awareness regarding CRC symptoms and risk factors.^20^ Although the level of education among our study population (63.5% had a diploma or higher degrees) was higher than that of the UK study (25.6%), this observation did not reflect on the level of CRC awareness. In fact, the level of education may not be associated with the level of health literacy.^17^ As raising awareness could help reduce cases, ensure earlier detection, and improve survival, the Department of Health in England published a list of ‘key messages’ for CRC including warning signs and risk factors associated with the disease; moreover, the cancer reform strategy, issued by the Department of Health in England which emphasized the importance of raising awareness of cancer early warning signs in the general population.^20^ The findings of one study revealed that lack of awareness was the most important barrier to CRC screening.^28^ Therefore, the lack of educational campaigns and awareness promotional strategies for increasing health literacy and CRC awareness could be the most probable reason. Accordingly, designing educational programs involving physicians and media is significant for improving CRC awareness.

 The most recognized warning sign was a lump in the abdomen. Along with our result, a lump in the abdomen was the most recognized warning sign in Jordan, Palestine, and Qatar.^17,29,30^ However, the least recognized warning sign was unexplained weight loss, followed by tiredness/anemia. A study in Kuala Lumpur demonstrated that tiredness/anemia and unexplained weight loss were the two most common clinical symptoms presented among CRC patients in Kuala Lumpur.^31^ As they are nonspecific CRC symptoms, it is difficult to differentiate them from other diseases in the public population. In addition, they could be delayed in seeking medical professional help then efforts for informing individuals about detectable warning signs of CRC are inevitable, especially for symptoms that are associated with several diseases, and distinction is challenging. Additionally, some of the warning signs such as weight loss are more strongly associated with a diagnosis of CRC and should be emphasized, particularly in older adults who are at most risk.^20^

 The most recognized risk factor was eating processed meat, which conforms to the results of a study performed in Qatar.^17^ Awareness of some lifestyle factors associated with CRC which are modifiable (e.g., physical activity, intake of red meat, and intake of fruit and vegetables) was particularly poor, which is in conformity with the results of other studies.^20,32^ Iranian population consumes a large amount of red meat in their usual diet compared to other meats, thus health promotion professionals should consider this fact in their educational interventions.^33^ As greater awareness could lead to increased healthy behaviors^32^ and thus could go some way towards decreasing the overall burden of diseases such as cancer on the population, health promotion initiatives to educate the public about the association between living a healthy lifestyle and cancer and increasing awareness of these risk factors are essential in ensuring a better understanding of the links between lifestyle and cancer.^20^ Most of the participants recognized drinking alcohol as a risk factor which may be consistent with Islamic beliefs in Iranian culture, where drinking alcohol is forbidden. Similarly, the majority of respondents in other Islamic countries agreed with the fact that alcohol consumption is harmful and could cause cancer.^34^ Current findings are consistent with the results of other studies which also represented how much work was required to enhance a better understanding of the links between lifestyle and CRC.^11,34^ Moreover, awareness of the role of physical activity in preventing CRC in the current study was low. Similarly, the national US survey also reported that a few respondents listed physical activity as a means of preventing CRC.^35^ Raising awareness of the protective effect of physical activity against CRC may encourage individuals to become more active. Efforts to raise this awareness should be undertaken within the context of comprehensive and collaborative interventions to promote physical activity. Between non-modifiable risk factors, diabetes was the least recognized risk factor, which is in line with the findings of other studies.^17,20,30,36^ The prevalence of diabetes in Iran has been increasing^13^; therefore, healthcare professionals should consider this issue to actively educate the public regarding the risk of CRC in diabetics and should focus more on the long-term consequences of diabetes. As the Iranian population trusts the healthcare professionals, they should take this opportunity for communicating messages for CRC prevention through lifestyle modification.

 Single participants had higher awareness of CRC warning signs. These results are consistent with those of another study,^6^ indicating that participants under 50 years old had higher awareness of CRC risk factors. The result of a US study also showed that participants above 50 years did not know about the protective effect of physical activity against CRC.^35^ The results of a study in the UK study also revealed that younger respondents were more aware of the link between CRC and older age than older respondents and had higher awareness of alcohol and bowel disease as risk factors for CRC, which could reflect better knowledge of CRC risk factors among younger adults more generally.^20^

 Based on our results, employment, higher education, and higher income were associated with a higher level of CRC warning signs and risk factor awareness, which corroborates with the findings of previous studies in Malaysia and the United Kingdom, reporting that participants from affluent groups demonstrated a higher level of cancer awareness.^6,20,36,37^ Furthermore, those who had CRC in their family showed a higher level of CRC warning signs and risk factor awareness. Having a family history of CRC could be more strongly associated with knowledge of risk factors due to increased perceptions of risk and motivations to prevent the disease.^20^ As individuals may have heard about CRC from their family, they are more familiar with the disease or friends, raising their awareness.^6^

 The results of logistic regression indicated that older age, male, and higher warning sign awareness are predictors of CRC screening awareness. In another study, older respondents represented higher knowledge of symptoms of CRC than younger respondents, which is encouraging because they are at higher risk and have a greater need to correctly identify symptoms and participate in screening programs. Men had higher awareness of the importance of weight and exercise as CRC risk factors. This could be because men are aware that lifestyle risk factors are more strongly associated with cancer in men than in women.^20^ Older persons and males are more vulnerable to CRC.^23^ It has been reported that lack of awareness was a barrier to screening adherence and can be a predictor of participation in CRC screening,^38,39^ while early diagnosis is one of the effective factors in the patient’s survival rate.^40^

 Similar to other studies, this study had some limitations. In the current study, information was collected using a self-administered questionnaire, thus recall and selection are the common biases in all such studies; although we tried our best to remove or decrease these biases, they can be considered the limitations of our study. The cross-sectional nature of the study is also one of the limitations of this study. On the other hand, one strength of this study was the large sample size of the public population. In addition, this was the first study using the validated Cancer Awareness Measurement questionnaire in Iran. The findings of our study can represent the level of CRC awareness among the population in a developing country. By using a standardized questionnaire, the international comparison of CRC awareness will be possible. Furthermore, in this study, samples participated from different age groups and both genders. Exploring CRC screening barriers in the Iranian population is suggested for future research.

**Highlights**

- Mean awareness of CRC warning signs and risk factors was low.
- Most of the participants (97.2%) had no awareness of the CRC screening program.
- Participants from more affluent groups had significantly higher CRC warning signs awareness.
- Participants from more affluent groups had significantly higher awareness of CRC risk factors.
- Awareness of age, gender, and warning signs was significantly associated with screening awareness.

## Conclusion

 Understanding Iranian adults’ awareness regarding CRC may provide valuable information for making public health policy decisions for primary prevention, screening, and improvement of survival from CRC. In general, the awareness of CRC symptoms, risk factors, and screening was low among the public population in Mashhad. Specifically, the regression analysis revealed that less affluent participants had low awareness of CRC symptoms and risk factors. This underlines the importance of tailoring future educational campaigns that are relevant and specific with a focus on those with low education and low income. The Ministry of Health of Iran should play a major role in developing national guidelines for CRC prevention and implementing its screening at least among high-risk groups.

## Acknowledgements

 This article is part of the PhD thesis in the field of Health Education and Health Promotion sponsored by Mashhad University of Medical Science and approved by the Ethics Committee of Mashhad University of Medical Sciences with the code of ethics IR.MUMS.FHMPM.REC.1400.008 (Code: 991804). The authors of this study express their sincere gratitude to all authorities of the Student Research Committee of Mashhad University of Medical Sciences and health care centers in Mashhad.

## Authors’ Contribution

**Conceptualization:** Samira Olyani, Nooshin Peyman, Hossein Ebrahimipour, Mehrsadat Mahdizadeh Taraghdari, Jamshid Jamali.

**Data curation:** Samira Olyani, Nooshin Peyman, Hossein Ebrahimipour, Mehrsadat Mahdizadeh Taraghdari.

**Formal analysis:** Samira Olyani, Nooshin Peyman, Jamshid Jamali.

**Funding acquisition:** Samira Olyani, Nooshin Peyman.

**Investigation:** Samira Olyani, Nooshin Peyman.

**Methodology:** Jamshid Jamali.

**Project administration:** Samira Olyani, Nooshin Peyman.

**Resources:** Samira Olyani, Nooshin Peyman.

**Software:** Samira Olyani, Nooshin Peyman.

**Supervision:** Samira Olyani, Nooshin Peyman, Hossein Ebrahimipour.

**Validation:** Samira Olyani, Nooshin Peyman, Mehrsadat Mahdizadeh Taraghdari.

**Visualization:** Samira Olyani, Nooshin Peyman.

**Writing–original draft:** Samira Olyani, Nooshin Peyman, Mehrsadat Mahdizadeh Taraghdari.

**Writing–review & editing:** Samira Olyani, Nooshin Peyman, Hossein Ebrahimipour, Jamshid Jamali.

## Competing Interests

 The authors declare that they have no competing interests.

## Funding

 No financial support was received for this study.

## References

[1] Hamza A, Argaw Z, Gela D (2021). Awareness of colorectal cancer and associated factors among adult patients in Jimma, South-West Ethiopia: an institution-based cross-sectional study. Cancer Control.

[2] GLOBOCAN. Estimated Number of Colorectal Cancer New Cases in 2020, Worldwide, Both Sexes, All Ages. Cancer Today. 2020. Available from: https://gco.iarc.fr/today/online-analysis-table? Updated December 2020. Accessed May 20, 2023.

[3] Onyoh EF, Hsu WF, Chang LC, Lee YC, Wu MS, Chiu HM (2019). The rise of colorectal cancer in Asia: epidemiology, screening, and management. Curr Gastroenterol Rep.

[4] Kocarnik JM, Compton K, Dean FE, Fu W, Gaw BL, Harvey JD (2022). Cancer incidence, mortality, years of life lost, years lived with disability, and disability-adjusted life years for 29 cancer groups from 2010 to 2019: a systematic analysis for the Global Burden of Disease Study 2019. JAMA Oncol.

[5] Roshandel G, Ferlay J, Ghanbari-Motlagh A, Partovipour E, Salavati F, Aryan K (2021). Cancer in Iran 2008 to 2025: recent incidence trends and short-term predictions of the future burden. Int J Cancer.

[6] Su TT, Goh JY, Tan J, Muhaimah AR, Pigeneswaren Y, Khairun NS (2013). Level of colorectal cancer awareness: a cross sectional exploratory study among multi-ethnic rural population in Malaysia. BMC Cancer.

[7] Hoseini B, Rahmatinejad Z, Goshayeshi L, Bergquist R, Golabpour A, Ghaffarzadegan K (2022). Colorectal cancer in North-Eastern Iran: a retrospective, comparative study of early-onset and late-onset cases based on data from the Iranian hereditary colorectal cancer registry. BMC Cancer.

[8] Al-Hajeili M, Abdulwassi HK, Alshadadi F, Alqurashi L, Idriss M, Halawani L (2019). Assessing knowledge on preventive colorectal cancer screening in Saudi Arabia: a cross-sectional study. J Family Med Prim Care.

[9] Imran M, Sayedalamin Z, Alsulami SS, Atta M, Baig M (2016). Knowledge and awareness of colorectal cancer among undergraduate students at King Abdulaziz University, Jeddah, Saudi Arabia: a survey-based study. Asian Pac J Cancer Prev.

[10] Elshami M, Ayyad MM, Alser M, Al-Slaibi I, Ahmed Naji S, Mohamad BM (2022). Awareness of colorectal cancer signs and symptoms: a national cross-sectional study from Palestine. BMC Public Health.

[11] Mozafar Saadati H, Okhovat B, Khodamoradi F (2021). Incidence and risk factors of colorectal cancer in the Iranian population: a systematic review. J Gastrointest Cancer.

[12] Mansour-Ghanaei A, Joukar F, Mansour-Ghanaei F, Rasoulian J, Naghipour MR, Fani A (2015). Knowledge about colorectal cancer in Northern Iran: a population-based telephone survey. Asian Pac J Cancer Prev.

[13] Khodakarami R, Abdi Z, Ahmadnezhad E, Sheidaei A, Asadi-Lari M (2022). Prevalence, awareness, treatment and control of diabetes among Iranian population: results of four national cross-sectional STEPwise approach to surveillance surveys. BMC Public Health.

[14] Consolo P, Luigiano C, Strangio G, Scaffidi MG, Giacobbe G, Di Giuseppe G (2008). Efficacy, risk factors and complications of endoscopic polypectomy: ten year experience at a single center. World J Gastroenterol.

[15] Magsoudlou M, Rakhshanderou S, Pourhoseingholi MA, Ghaffari M (2021). Knowledge and health beliefs of staff of Shahid Beheshti University of Medical Sciences, Tehran, Iran, on colorectal cancer and related factors: a cross-sectional research based on health belief model. J Health Syst Res.

[16] Al Abdouli L, Dalmook H, Akram Abdo M, Carrick FR, Abdul Rahman M (2018). Colorectal cancer risk awareness and screening uptake among adults in the United Arab Emirates. Asian Pac J Cancer Prev.

[17] Al-Dahshan A, Chehab M, Bala M, Omer M, AlMohamed O, Al-Kubaisi N (2020). Colorectal cancer awareness and its predictors among adults aged 50-74 years attending primary healthcare in the state of Qatar: a cross-sectional study. BMJ Open.

[18] Taha H, Jaghbeer MA, Shteiwi M, AlKhaldi S, Berggren V (2015). Knowledge and perceptions about colorectal cancer in Jordan. Asian Pac J Cancer Prev.

[19] Tfaily MA, Naamani D, Kassir A, Sleiman S, Ouattara M, Moacdieh MP (2019). Awareness of colorectal cancer and attitudes towards its screening guidelines in Lebanon. Ann Glob Health.

[20] Power E, Simon A, Juszczyk D, Hiom S, Wardle J (2011). Assessing awareness of colorectal cancer symptoms: measure development and results from a population survey in the UK. BMC Cancer.

[21] Heinze G, Dunkler D (2017). Five myths about variable selection. Transpl Int.

[22] Koo JH, Leong RW, Ching J, Yeoh KG, Wu DC, Murdani A (2012). Knowledge of, attitudes toward, and barriers to participation of colorectal cancer screening tests in the Asia-Pacific region: a multicenter study. Gastrointest Endosc.

[23] Nasaif HA, Al Qallaf SM (2018). Knowledge of colorectal cancer symptoms and risk factors in the Kingdom of Bahrain: a cross- sectional study. Asian Pac J Cancer Prev.

[24] Knudsen MD, Hoff G, Tidemann-Andersen I, Bodin GE, Øvervold S, Berstad P (2021). Public awareness and perceptions of colorectal cancer prevention: a cross-sectional survey. J Cancer Educ.

[25] Kaya O, Hoca O, Kulaçoğlu H (2013). Knowledge and awareness of auxiliary health personnel about colorectal cancer. Turk J Gastroenterol.

[26] Brandt HM, Dolinger HR, Sharpe PA, Hardin JW, Berger FG (2012). Relationship of colorectal cancer awareness and knowledge with colorectal cancer screening. Colorectal Cancer.

[27] Anderson AS, Caswell S, Macleod M, Craigie AM, Stead M, Steele RJ (2015). Awareness of lifestyle and colorectal cancer risk: findings from the BeWEL study. Biomed Res Int.

[28] Bidouei F, Abdolhosseini S, Jafarzadeh N, Izanloo A, Ghaffarzadehgan K, Abdolhosseini A (2014). Knowledge and perception toward colorectal cancer screening in east of Iran. Int J Health Policy Manag.

[29] Mhaidat NM, Al-Husein BA, Alzoubi KH, Hatamleh DI, Khader Y, Matalqah S (2018). Knowledge and awareness of colorectal cancer early warning signs and risk factors among university students in Jordan. J Cancer Educ.

[30] Elshami M, Dwikat MF, Al-Slaibi I, Alser M, Mohamad BM, Isleem WS (2022). Awareness of colorectal cancer risk factors in Palestine: where do we stand?. JCO Glob Oncol.

[31] Rashid MR, Aziz AF, Ahmad S, Shah SA, Sagap I (2009). Colorectal cancer patients in a tertiary referral centre in Malaysia: a five year follow-up review. Asian Pac J Cancer Prev.

[32] Hawkins NA, Berkowitz Z, Peipins LA (2010). What does the public know about preventing cancer? Results from the Health Information National Trends Survey (HINTS). Health Educ Behav.

[33] Khodayari S, Sadeghi O, Safabakhsh M, Mozaffari-Khosravi H (2022). Meat consumption and the risk of general and central obesity: the Shahedieh study. BMC Res Notes.

[34] Al-Azri M, Al-Hamedi I, Al-Awisi H, Al-Hinai M, Davidson R (2015). Public awareness of warning signs and symptoms of cancer in Oman: a community-based survey of adults. Asian Pac J Cancer Prev.

[35] Coups EJ, Hay J, Ford JS (2008). Awareness of the role of physical activity in colon cancer prevention. Patient Educ Couns.

[36] Sindhu CK, Nijar AK, Leong PY, Li ZQ, Hong CY, Malar L (2019). Awareness of colorectal cancer among the urban population in the Klang Valley. Malays Fam Physician.

[37] Robb K, Stubbings S, Ramirez A, Macleod U, Austoker J, Waller J (2009). Public awareness of cancer in Britain: a population-based survey of adults. Br J Cancer.

[38] Gimeno Garcia AZ, Hernandez Alvarez Buylla N, Nicolas-Perez D, Quintero E (2014). Public awareness of colorectal cancer screening: knowledge, attitudes, and interventions for increasing screening uptake. ISRN Oncol.

[39] Gimeno-García AZ, Quintero E, Nicolás-Pérez D, Jiménez-Sosa A (2011). Public awareness of colorectal cancer and screening in a Spanish population. Public Health.

[40] Roshani D, Moradi G, Rasouli MA (2023). Survival analysis of patients with colorectal cancer undergoing combined treatment: a retrospective cohort study. J Res Health Sci.

